# Membrane-Bound IL-21 Promotes Sustained *Ex Vivo* Proliferation of Human Natural Killer Cells

**DOI:** 10.1371/journal.pone.0030264

**Published:** 2012-01-18

**Authors:** Cecele J. Denman, Vladimir V. Senyukov, Srinivas S. Somanchi, Prasad V. Phatarpekar, Lisa M. Kopp, Jennifer L. Johnson, Harjeet Singh, Lenka Hurton, Sourindra N. Maiti, M. Helen Huls, Richard E. Champlin, Laurence J. N. Cooper, Dean A. Lee

**Affiliations:** 1 Division of Pediatrics, MD Anderson Cancer Center, The University of Texas, Houston, Texas, United States of America; 2 Graduate School of Biomedical Sciences, Health Science Center, The University of Texas, Houston, Texas, United States of America; 3 Department of Stem Cell Transplantation and Cellular Therapy, MD Anderson Cancer Center, The University of Texas, Houston, Texas, United States of America; University of Palermo, Italy

## Abstract

NK cells have therapeutic potential for a wide variety of human malignancies. However, because NK cells expand poorly *in vitro*, have limited life spans *in viv*o, and represent a small fraction of peripheral white blood cells, obtaining sufficient cell numbers is the major obstacle for NK-cell immunotherapy. Genetically-engineered artificial antigen-presenting cells (aAPCs) expressing membrane-bound IL-15 (mbIL15) have been used to propagate clinical-grade NK cells for human trials of adoptive immunotherapy, but *ex vivo* proliferation has been limited by telomere shortening. We developed K562-based aAPCs with membrane-bound IL-21 (mbIL21) and assessed their ability to support human NK-cell proliferation. In contrast to mbIL15, mbIL21-expressing aAPCs promoted log-phase NK cell expansion without evidence of senescence for up to 6 weeks of culture. By day 21, parallel expansion of NK cells from 22 donors demonstrated a mean 47,967-fold expansion (median 31,747) when co-cultured with aAPCs expressing mbIL21 compared to 825-fold expansion (median 325) with mbIL15. Despite the significant increase in proliferation, mbIL21-expanded NK cells also showed a significant increase in telomere length compared to freshly obtained NK cells, suggesting a possible mechanism for their sustained proliferation. NK cells expanded with mbIL21 were similar in phenotype and cytotoxicity to those expanded with mbIL15, with retained donor KIR repertoires and high expression of NCRs, CD16, and NKG2D, but had superior cytokine secretion. The mbIL21-expanded NK cells showed increased transcription of the activating receptor CD160, but otherwise had remarkably similar mRNA expression profiles of the 96 genes assessed. mbIL21-expanded NK cells had significant cytotoxicity against all tumor cell lines tested, retained responsiveness to inhibitory KIR ligands, and demonstrated enhanced killing via antibody-dependent cell cytotoxicity. Thus, aAPCs expressing mbIL21 promote improved proliferation of human NK cells with longer telomeres and less senescence, supporting their clinical use in propagating NK cells for adoptive immunotherapy.

## Introduction

NK cells are potent effectors of the innate immune system [1] with cytotoxic and immunoregulatory function [2], [3]. Human NK cells are typically characterized as lymphocytes (CD2^pos^) expressing CD56 or CD16 and lacking CD3 expression [4], and make up from 1–32.6% of peripheral blood lymphocytes in normal subjects [5]. Recently, NKp46 has been suggested as a unifying marker of NK cells across species [6]. Unlike T-cells, NK cells recognize targets in a major histocompatibility complex (MHC)-unrestricted manner. NK cells display a variety of activating receptors, including NKG2D and the natural cytotoxicity receptors NKp30, NKp44, NKp46, whose activation signals compete with inhibitory signals provided primarily by killer immunoglobulin receptors (KIR) and CD94/NKG2A. NK cells play an important role in initiating responses to infection, including infections of importance in the peri-transplant setting such as cytomegalovirus (CMV), herpes simplex virus (HSV), respiratory syncitial virus (RSV), and influenza. Donor KIR-mismatched NK cells can suppress recipient derived lymphocytes, reducing the risk of rejection, and react against recipient dendritic cells [7], thereby reducing the allostimulous for GvHD [8].

With antiviral, anti-GvH, and anti-cancer potential, adoptive immunotherapy with natural killer (NK) cells has emerged as promising anti-cancer treatment. NK cells have therapeutic potential for a wide variety of human malignancies, including sarcomas [9], [10], myeloma [11], carcinomas [12], [13], [14], [15], lymphomas [16], and leukemias [14], [17], [18]. Until recently, the clinical efficacy and effective application of NK cell immunotherapy has been limited by the inability to obtain sufficient cell numbers for adoptive transfer, as these cells represent a small fraction of peripheral white blood cells, expand poorly *ex vivo*, and have limited life spans *in vivo*. The ability to harvest large numbers of peripheral blood lymphocytes through leukapheresis, deplete alloreactive T cells, and activate the remaining NK cells with IL-2 has enabled NK cell adoptive immunotherapy, but this process is expensive, invasive, and remains limited in cell dose to a single infusion of typically less than 2×10^7^ NK cells/kg [19]. Using this approach, Miller *et al* [14] demonstrated that infusion of haploidentical NK cells after chemotherapy could induce remission in poor-prognosis AML patients, and remission was associated with KIR mismatch. In a similar study, Rubnitz *et al* [18] reported the safety of KIR-mismatched NK cell infusion as post-remission consolidation therapy for children with AML, with no relapses reported in the 10 patients treated. A similar approach has been used for adoptive transfer of NK cells in patients with refractory lymphoma [16] and multiple myeloma [20]. GvHD was not reported in any of these studies.

Identifying the optimal signal for propagation of NK cells *in vitro* has been problematic due in part to the large number of activating and inhibitory receptors, cooperative receptor pairs, and overlapping signaling pathways involved in maturation, activation, and proliferation. Expansion of donor NK cells has been reported with various combinations of cytokines [17], [21], [22], [23], [24], [25], [26], [27], [28], cytokine fusion proteins [29], [30], cytokines and OKT3 [11], [12], [31], [32], [33], [34], cytokines and stromal support [35], antibody-coated beads [36], bisphosphonate-capped dendrimers [37], methyl-β-cyclodextrin [38], or feeder cells derived from EBV-lymphoblastoid cell lines [39], [40], [41], [42], [43] or K562 [44], [45], [46], [47]. K562-based aAPCs transduced with 4-1BBL (CD137L) and membrane-bound IL-15 (mbIL15) [45] promoted a mean NK-cell expansion of 277-fold in 21 days, but continued proliferation was limited by senescence attributed to telomere shortening. K562 expressing CD137L, MICA, and soluble IL-15 yielded a mean NK cell expansion of 550-fold in 24 days [46]. The highest doses of NK cells yet reported to be infused into patients were derived by expanding autologous CD3-depleted pheresis products for three weeks with IL-2, OKT3, and irradiated PBMC feeder cells [48]. With the development of methods to expand primary human NK cells *in vitro*, there is now renewed interest in NK cell immunotherapy [13], [22], [26], [41], [45].

Common γ-chain cytokines, of which IL-2, IL-15 and IL-21 are members, are important in NK cell activation, maturation, and proliferation. IL-15 has a well-recognized role in maturation, survival, and homeostatic expansion of NK cells. The discovery of IL-21 was linked to its role in proliferation and maturation of NK cells [49], but subsequent studies have been widely disparate, identifying it as both activating and suppressive [50], [51], [52]. Although soluble IL-21 alone does not induce significant proliferation of mature murine NK cells and IL-21R knockout mice have normal NK cell numbers [50], IL-21 synergizes with IL-2, IL-15, and Flt-3L in the generation of NK cells from bone marrow [49] and cord blood [49], [53], [54], [55], [56]. IL-21 may activate NK cell lytic activity through upregulation of costimulatory receptors, perforin, and granzyme [57]. To exploit this potential of these cytokines, we developed membrane-bound chimeras of IL-21 (mbIL21) and IL-15 (mbIL15), and investigated NK cell expansion, phenotype, and function in response to K562 aAPCs genetically modified with mbIL21 and/or mbIL15.

## Materials and Methods

### Ethics Statement

All work with human samples was performed under protocols approved by the Institutional Review Board of MDACC and/or BCM. Written informed consent to participation in an approved protocol was obtained from all participants (or next of kin/legal guardian) prior to collection of samples, with the exception of such cases in which the requirement for consent was waived by the IRB (e.g., anonymized buffy coats from the local blood bank or commercially available human cell lines). Human NK cells were purified or expanded from anonymized blood bank buffy coats obtained under this IRB-approved protocol. Human tumor xenografts were established according to protocol 05-07-04832 approved by the Institutional Animal Care and Use Committee of MDACC.

### Cells and cell lines

Anonymized normal human donor buffy coats were obtained from the Gulf Coast Regional Blood Center (Houston, TX).

**Figure 1:**
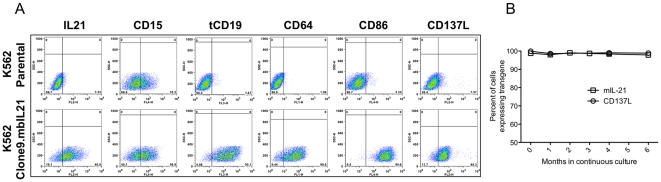
Surface expression of CD15 and transgene receptors on Clone 9.mbIL21 aAPC, and stability of expression of relevant receptors. After limiting-dilution cloning, cloned aAPCs were assessed for expression of transgenes, *A*. Since CD15 expression was variable among the clones and is felt to play a role in NK cell activation, its expression was also assessed. After selection of a clone with expression of all surface proteins, the clone was maintained in culture for 6 months, with periodic re-evaluation of CD137L and mbIL21 expression, *B*.

721.221 cell lines, including those genetically-modified to express selected KIR ligands, were obtained as previously described [58]. The parental K562 cell line was obtained from the American Type Culture Collection (ATCC). K562 aAPCs (Table 1) [59] were produced by retroviral transduction with CD64 (FcγRI), CD86 (B7-2), CD137L (4-1BBL), and truncated CD19 to create the core aAPC designated Clone 9, or with the same four core transgenes and mbIL15 to create the aAPC designated Clone 4, as described previously [60]. Clone D2 and Clone 9.mbIL21 were created by transducing Clone 4 and Clone 9, respectively, with a *Sleeping Beauty* transposon expressing mbIL21 as described previously [61]. Clone 27 was created by transducing Clone 9 with a *Sleeping Beauty* transposon expressing IL-15 fused to IL-15Rα with a linker. aAPC clones were selected for high transgene expression after single-cell cloning (Clone 9.mbiL21 shown in Figure 1A), except aAPC Clone D2 which was selected for high transgene expression by high-speed flow sorting.

**Table 1:** Artificial APCs used in this study.

aAPC name	Cytokine transgene
Clone 9	None
Clone 4	Membrane-bound IL-15 mutein (mbIL15)
Clone 27	IL-15 fused to IL-15Rα with linker
Clone 9.mbIL21	Membrane-bound IL-21 mutein (mbIL21)
Clone D2	mbIL15 and mbIL21

All aAPCs include the transgenes for tCD19, CD64, CD86, and CD137L.

AML10 human leukemic myeloblasts were obtained from the peripheral blood of a newly-diagnosed pediatric patient after written consent was obtained under an IRB-approved collection and banking protocol. AML10 cells were expanded by serial passage in NOD.Cg-*Rag1^tm1Mom^Prf1^tm1Sdz^*/SzJ mice (Jackson Laboratories) [62], and verified >98% human CD33^pos^ after recovery. Raji and ARH-77 were obtained from ATCC. CHP134 and SK-N-BE(2) were obtained from the COG Cell Culture and Xenograft Repository. U937 was a gift from T. Horton of Baylor College of Medicine, Houston TX (BCM). SAOS-2, HCT15, and SK-MEL-37 were obtained from E. Kleinerman, D. Hughes, and L. Radvanyi, respectively, of the University of Texas MD Anderson Cancer Center (MDACC).

All cell lines were validated by STR DNA fingerprinting using the AmpFℓSTR Identifiler kit according to manufacturer instructions (Applied Biosystems cat 4322288). The STR profiles were compared to fingerprint data on the ATCC fingerprint database (ATCC.org), the Cell Line Integrated Molecular Authentication database (CLIMA) version 0.1.200808 (bioinformatics.istge.it/clima/), the COG Cell Line and Xenograft STR Database (strdb.cogcell.com/), and the MD Anderson fingerprint database. The STR profiles were last performed on October 15, 2010 and either matched to their known DNA fingerprints, or were unique (AML10 and AML13).

### Antibodies

Murine anti-human NKp30, NKp44, NKp46, CD16, CD56, NKG2D, isotype control mAb, and the mouse IgG2a anti-disialoganglioside GD2 (clone 14.G2a) were obtained from BD Biosciences (Bedford, MA). Murine anti-human KIR2DL2/3 was obtained from Miltenyi (Auburn, CA). Murine anti-human KIR2DL1 and KIR3DL1 were obtained from R&D Systems (Minneapolis, MN). The humanized IgG1 anti-human CD20 (rituximab, Genentech) was purchased through the institutional pharmacy.

### NK cell purification by negative selection

NK cells were enriched from buffy coats with RosetteSep Human NK Cell Enrichment Cocktail (StemCell Technologies, Inc., Vancouver, BC, Canada), using 10 µL per 1 mL of buffy coat. When further purification was needed to achieve NK cell purity to ≥95% (as assessed by flow cytometry as CD3^neg^CD56^pos^), or for depletion of T cells during NK cell expansion, the method of Warren *et al.* was used [63], in which 3rd party RBCs were added to enhance agglutination and depletion of unwanted cells.

### Ex vivo expansion of human NK cells

**Figure 2:**
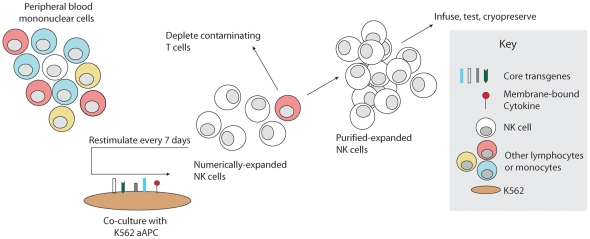
Schema for NK cell manufacturing with aAPCs. Artificial antigen-presenting cells (aAPCs) were produced by genetic modification of K562 to express costimulatory molecules and membrane-bound cytokines. To expand NK cells *ex* vivo, unfractionated PBMC are stimulated weekly with irradiated PBMC, inducing rapid proliferation of NK cells and in some cases non-specific expansion of T cells. Contaminating T cells may be depleted, and the remaining purified NK cells may be stimulated weekly by the aAPCs as needed to obtain sufficient numbers. Expanded NK cells may be used directly or cryopreserved for future use.

**Figure 3:**
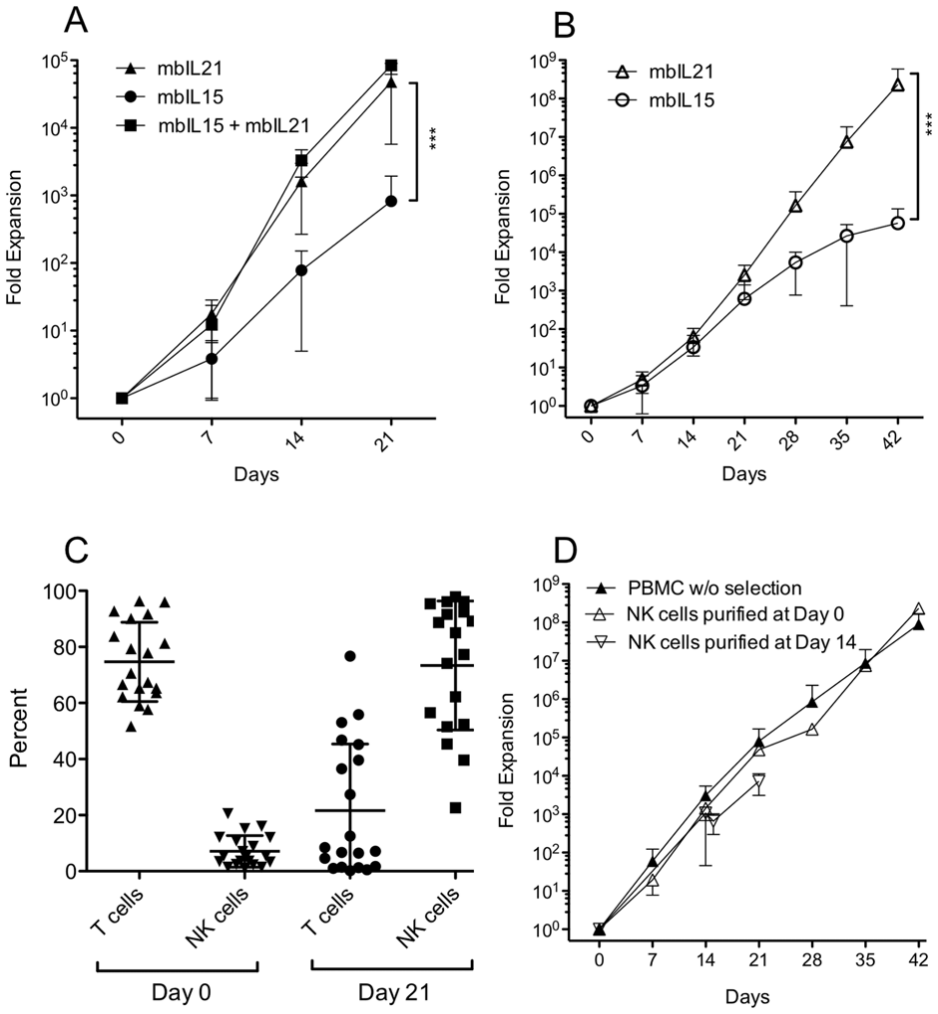
NK cell expansion and purity after repeated weekly stimulation with aAPCs expressing membrane-bound cytokines. *A*, Mean expansion of CD3^neg^/CD16-or-56^pos^ NK cells from 22 donors expanded for 21 days using aAPCs bearing mbIL15 or mbIL21. NK cells from five donors were also expanded with aAPCs expressing both mbIL15 and mbIL21. *B*, Mean expansion of NK cells from 4 donors expanded for 42 days using aAPCs bearing mbIL15 or mbIL21. *C*, Percent of CD3^pos^ T/NKT cells and CD3^neg^/CD16-or-56^pos^ NK cells in the starting PBMC product (day 0) and at the end of 21 days of expansion on Clone 9.mbIL21 from 20 donors. *D*, Mean expansion of CD3^neg^/CD16-or-56^pos^ NK cells on Clone 9.mbIL21 from unfractionated PBMC (n = 19), unfractionated PBMC followed by NK purification at day 14 of expansion (n = 3), or from NK cells purified from PBMC at day 0 prior to expansion (n = 13). Mean +/− SD is shown for each plot. All p values indicated are for *t-*test of fold expansion at the end of the expansion period.

NK cells were cultured in NK cell media consisting of RPMI 1640 (Cellgro/Mediatech, Manassas, VA) supplemented with 50 IU/ml recombinant human IL-2 (Proleukin, Novartis Vaccines and Diagnostics, Inc), 10% Fetal Bovine Serum (Invitrogen, Carlsbad, CA), L-glutamine (Gibco/Invitrogen, Carlsbad, CA), and penicillin/streptomycin (Cellgro/Mediatech, Manassas, VA). NK cells were expanded from PBMC as previously described (Figure 2) [64]. Briefly, peripheral blood mononuclear cells (PBMC) were first isolated from buffy coats using Ficoll-Paque Plus (GE HealthCare, Piscataway, NJ). PBMC were co-cultured in T-75 flasks (Corning, Corning, NY) with irradiated (100 cGy) K562 aAPCs at a ratio of 1∶2 (PBMC∶aAPC) in NK cell media at 2×10^5^ PBMC/mL. Cultures were refreshed with half-volume media changes every two to three days, and re-stimulated with aAPCs at ratio of 1∶1 every seven days. When necessary, a portion of the expanding cells was carried forward for subsequent stimulations, and the remaining cells cryopreserved. NK cell expansion was calculated from the resulting cultures as if all cells were carried forward in the expansion. Where indicated in Figure 3B and D, expansion cultures were initiated from purified NK cells instead of unfractionated PBMC, or T cells were depleted from the expansion culture prior to the 3rd stimulation at day 14.

### Flow cytometry

For direct surface staining, cells were incubated with indicated antibodies for 30 min at 4°C, washed, and resuspended in staining buffer. Data were acquired using a FACSCalibur cytometer (BD Biosciences) and analyzed using FlowJo software (Tree Star, Inc., Ashland, OR). The expanding cell subpopulations (T/NKT (CD3^pos^) and NK (CD3^neg^ and CD16-or-56^pos^)) and NK cell phenotypes were determined weekly during expansion.

### Cytotoxicity assay

NK cell cytotoxicity was determined using the calcein release assay, a fluorometric assay comparable to the chromium release assay in determining NK cell cytotoxicity [65], [66], as described [64]. Target cells were labeled with 0.5–5 µg/mL (titrated for each target cell line) of calcein-AM (Sigma-Aldrich) for 1 h at 37 °C with occasional shaking. Cells were co-cultured at the indicated effector-to-target (E∶T) ratios and incubated at 37 °C for 4 h. After incubation, 100 µL of the supernatant was harvested and transferred to a new plate. Absorbance at 570 nm was determined using a SpectraMax Plus^384^ spectrophotometer. The percent lysis was calculated according to the formula [(experimental release− spontaneous release)/(maximum release−spontaneous release)]×100. For ADCC experiments, antibodies were added to the lysis culture at 1 µg/mL. Video fluorescent microscopy of NK cells lysing calcein-loaded neuroblastoma targets was performed at a 5∶1 E∶T ratio using the Nikon BioStation, with images obtained every 4 minutes. 

### Telomere length analysis

NK cells were purified from buffy coat by the RosetteSep method, and an aliquot of the NK cells viably frozen. The remaining NK cells were cocultured with irradiated aAPCs for 7 days in NK cell media as described above. At the end of one week the expanded NK cells were frozen. Matched sets of expanded and unexpanded NK cells were thawed and assayed for telomere length using the reference cell line CEM-1301 as previously described [67] with minor modification. Briefly, 10^6^ cells were resuspended in hybridization buffer containing 75% deionized formamide (Spectrum Chemicals), 20 mM Tris, pH 7.0, 1% BSA, with or without 0.3 µg/ml telomere-specific FITC conjugated (C3TA2)3 PNA probe (Panagene). Samples were prepared in duplicate and subjected to heat denaturation of DNA for 15 min at 87°C followed by hybridization for 3 h at RT. Cells were then washed three times with 1 ml of wash buffer containing 70% formamide, 10% 10 mM Tris, 0.1% BSA, and 0.1% Tween 20 and once with 5% dextrose containing 10 mM Hepes, 0.1% BSA, and 0.1% Tween 20. After the last wash cells were resuspended in PBS containing 0.1% BSA, 10 µg/ml RNAse A (Cayman Chemical) and 0.06 µg/ml propidium iodide (BD Biosciences), incubated 2–3 h at room temperature and analyzed on a FACSCalibur flow cytometer (BD Biosciences). FITC-labeled fluorescent calibration beads (Quantum™ FITC MESF, Bangs Laboratories) were used for daily calibration of the FACS machine. Relative telomere length (RTL) was determined using CEM-1301 reference cells, calculated as: RTL = (MFI_sample cells with probe_−MFI_sample cells without probe_)/(MFI_reference cells with probe_−MFI_reference cells without probe_). The RTL of expanded NK cells was compared directly with same-donor unexpanded cells and reported as percent change.

### Determination of cytokine secretion by NK cells

NK cells were co-cultured overnight with parental K562 at a 1∶1 ratio. Supernatants were collected and concentrations of TNF-α, IFN-γ, and IL-6 were simulataneously determined using microparticle-based cytokine capture on Cytometric Bead Array (CBA) kits (BD Biosciences). Concentrations were calculated from mean fluorescent intensity based on standard curves and formulas provided with the kit.

### nCounter digital multi-plexed gene expression analysis

Gene expression in NK cells stimulated with mbIL15 or mbIL21 was assessed using the nCounter platform (nanoString Technologies, Inc., Seattle, WA) [68]. Purified NK cells from four donors were stimulated for one week in parallel expansions with either Clone 4 (mbIL15) or Clone 9.mbIL21). Total RNA was purified from each sample and assessed for expression of 96 genes. Gene expression was normalized to LDH (mean 6,076 copies detected from 100 ng of loaded mRNA), and plotted as mean ± SEM. Genes with borderline detection (≤10 normalized transcripts detected) were excluded from further analyses. Genes having ≥2-fold difference in mean expression between mbIL21 and mbIL15 culture were then identified.

### Statistics

Results are expressed as the mean ± SD or mean ± SEM as indicated. Student's *t* test was performed for pair-wise comparisons. Cytotoxicity across E∶T ratios were compared between groups using Two-way Repeated Measures ANOVA. One-way ANOVA with Dunnett's multiple comparisons test was applied to telomere lengths after stimulation with Clone 4 (mbIL15) as compared to other aAPCs. Statistical analysis was performed using Prism 5 for Mac Os X, version 5.0c (GraphPad Software, La Jolla, CA). *P* values less than 0.05 (*), less than 0.01 (**), less than 0.001 (***), or less than 0.0001 (****) were considered significant.

## Results

### mbIL21 supports improved proliferation of NK cells compared to mbIL15

We developed aAPCs for expansion of antigen-specific T cells by lentiviral transduction of the K562 cell line with CD64, CD86, CD137L, and truncated CD19, with (Clone 9) or without (Clone 4) mbIL15 [61], [69]. Based on a potential role of IL-21 in T-cell and NK-cell maturation and proliferation, we then developed mbIL21 [61] and used *Sleeping Beauty* transposition to produce derivative aAPCs bearing an IL-15∶IL15Rα fusion (Clone 27), mbIL21 (Clone 9.mbIL21), or both mbIL15 and mbIL21 (Clone D2) (Table 1). Typical transgene expression of Clone 9.mbIL21 is shown (Figure 1A), and we confirmed stability of the transgenes during 6 months of continuous culture (Figure 1B).

Using Clone 4, Clone 9.mbIL21, and Clone D2, we compared the expansion of NK cells in response to recursive stimulation by these APCs (Figure 2), normalizing the expansion to the NK cell content of each starting PBMC product. From PBMCs of 22 donors expanded in parallel with Clone 4 and Clone 9.mbIL2, we found that mbIL21 resulted in significantly greater fold expansion at 3 weeks (47,967+/−42,230, mean +/− SD) than mbIL15 (825+/−1108, mean +/− SD) (Figure 3A). PBMCs from four of these donors were also expanded with Clone D2 aAPCs expression both of these membrane-bound cytokines, which did not yield significantly greater expansion than mbIL21 alone (Figure 3A).

Since expansion of NK cells on aAPC expressing IL15 had previously been shown to be limited by telomere-induced senescence, we then initiated expansion of purified NK cells from four additional donors with Clone 4 (mbIL15) and Clone 9.mbIL21, and continued the expansion for 6 weeks to determine whether mbIL21-expanded NK cells would also undergo senescence. We found that mbIL15-supported NK cells slowed in their proliferative rate at 4–6 weeks as previously reported, whereas mbIL21-supported NK cells did not (Figure 3B).

### Expansion of NK cells from PBMC induces donor-dependent non-specific T-cell expansion

We noted that both Clone 4 and Clone 9.mbIL21 also stimulated significant non-specific T-cell expansion when PBMC were used as the starting product for NK-cell expansion. Among 20 NK-cell expansions with mbIL21 starting from an unfractionated PBMC product, we found that with successive stimulations during the culture period NK cells gradually became the predominant cell type with a relative loss of T cells (Figure 3C), as has been noted with other methods of NK-cell expansion [11]. Although NK cells rapidly increased in percentage for all donors over the first two weeks, this differential proliferation of NK cells tapered thereafter, resulting in a mean T-cell contamination of 21.7% (median 7.8%) by day 21. Although there was wide inter-donor variation, it was apparent that T-cell depletion would be required if the expanded products were to be given clinically in the setting of allogeneic adoptive transfer.

Since the expansion of purified NK cells (Figure 3B) appeared to be delayed during the first week compared to those expanded from PBMC (Figure 3A), we then determined whether NK cell expansion was affected by T-cell depletion in order to determine when it would be best performed to produce a clinically-viable product. Compared with NK cell proliferation in T-cell replete cultures, T-cell depletion prior to NK cell expansion or on day 14 just prior to the third stimulation resulted in no difference in proliferative rates.

### NK-cell expansion supported by mbIL15 or mbIL21 results in similar expression of activating and inhibitory receptors

**Figure 4:**
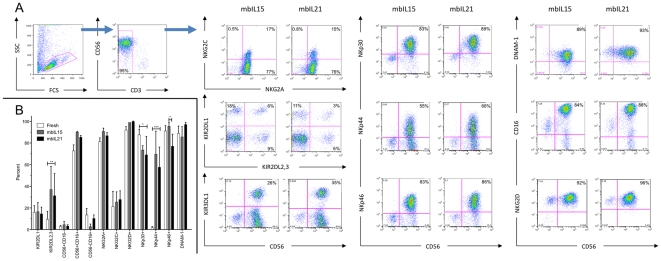
Phenotype of NK cells expanded on aAPCs bearing membrane-bound cytokines. NK cells purified from PBMC were stimulated weekly with either Clone 4 (mbIL15) or Clone 9.mbIL21 for 3 weeks. Expression of NK cell receptors was determined by flow cytometry. A) Representative dot plots of NK cells expanded from the same donor on Clone 4 (mbIL15) or Clone 9.mbIL21. B) Component subpopulations of fresh NK cells or NK cells expanded on Clone 4 (mbIL15) or Clone 9.mbIL21 from 4 donors as determined by flow cytometry. Mean +/− SD is shown. P values are for 2-way repeated-measures ANOVA comparing against mbIL21-expanded NK cells with Bonferroni correction (all significant P values are indicated).

Because IL-15 and IL-21 signal primarily through different STAT family molecules (STAT5 vs. STAT3, respectively), we questioned whether expansion with mbIL15 and mbIL21 might result in different NK-cell phenotypes, and assessed surface expression of the major NK-cell receptors on expanded NK cells. Although there was variation between donors, particularly in expression of KIR, Clone 4- and Clone 9.mbIL21-expanded NK cells were similar in both the percentage of positive cells and the relative receptor expression (MFI) for the receptors assessed (Figure 4A), with a slight decrease in NKp46^pos^ cells and a non-statistical trend towards increased number of CD56^neg^CD16^pos^ cells (Figure 4B). NK cells expanded by both methods were primarily CD16^bright^CD56^pos^ and expressed high levels of natural cytotoxicity receptors and KIR. When compared with freshly-isolated NK cells, mbIL21-expanded NK cells were similar to mbIL15-expanded NK cells in increased numbers of cells positive for KIR2DL2,3 and NKp44, and slightly decreased in NKp30^pos^ cells (Figure 4B).

### NK-cell expansion supported by mbIL15 or mbIL21 results in similar expression of immune-related genes

**Figure 5:**
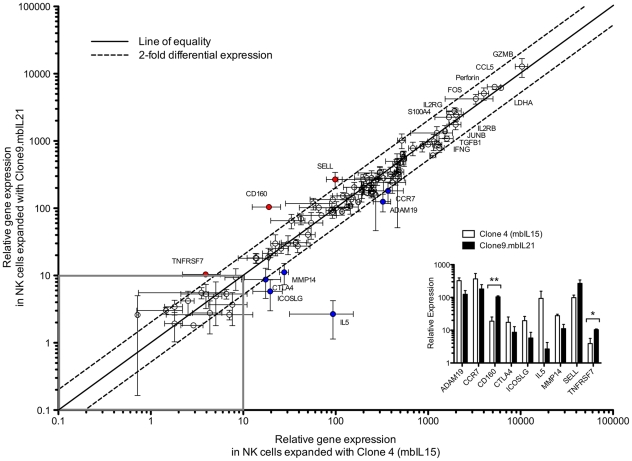
Gene expression in NK cells stimulated with mbIL15 or mbIL21 as assessed using the nCounter platform. Paired aliquots of purified NK cells from 4 donors were stimulated for one week with either Clone 4 (mbIL15) or Clone 9.mbIL21. Total RNA was purified and equal quantities hybridized for detection of expression of 96 genes. Gene expression was normalized to LDH (mean 6,076 copies detected), and the remaining data for each gene plotted as mean +/− SEM for each expansion condition. Genes with detection below background (set at ≤10 detected copies) were excluded as not biologically significant (grey box). The ten highest-expressed genes in addition to LDH are labeled, as are genes that are differentially expressed by >2 fold (red) or <0.5 fold (blue) in mbIL21-expanded cells. Differentially expressed genes were replotted (inset) for comparison, and two-tailed *t-*test applied.

We applied the nCounter technology for multiplex measurement of RNA [68] to accurately identify subtle changes in transcriptional profiles induced by mbIL21 as compared to mbIL15. We used a 96-gene panel initially developed for evaluating gene expression in T cells propagated on K562 aAPCs, and which assesses a broad variety of signaling, cytokine, chemokine, and cytolysis-related genes (Table S1). We found very high correlation between NK cells propagated with the support of mbIL15 and mbIL21 (Figure 5), with only 9 genes showing more than 2-fold differential expression between the two conditions. Only one of these, CD160, reached statistical significance between donor replicates when corrected for multiple comparisons (Figure 5, inset). Of note, the top ten genes expressed in these cells (other than LDH) were GZMB, PRF, FOS, CCL5, JUNB, IL2RG, IL2RB, TGFB1, IFNG, and S100A4, all associated with increased cell proliferation, activation, or known NK-cell effector function.

### NK-cell expansion with mbIL21 results in a significant increase in telomere length compared to mbIL15

**Figure 6:**
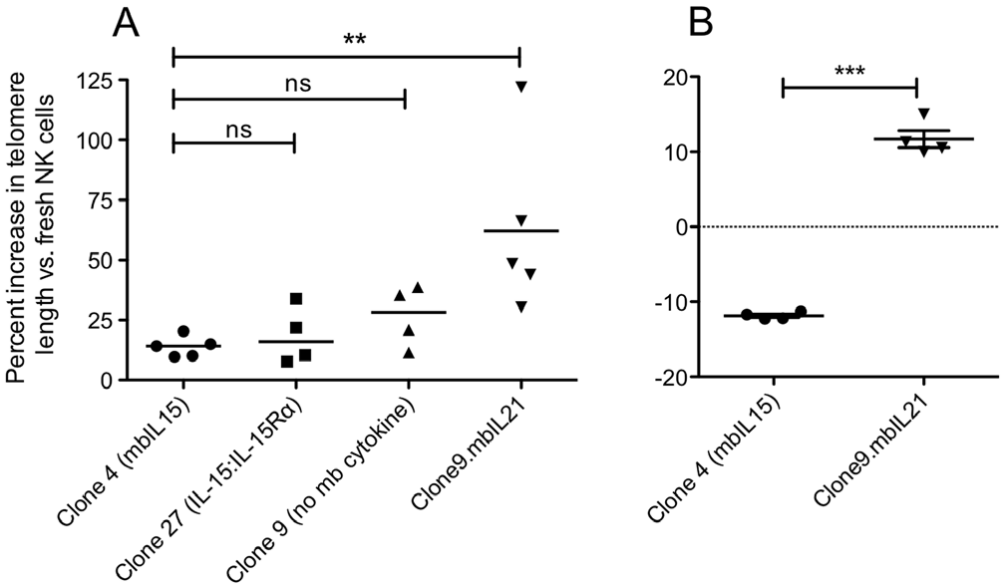
Change in relative telomere length of purified NK cells after expansion on aAPCs with or without membrane-bound cytokines. NK cells were purified from normal donor buffy coats. An aliquot was viably frozen for later comparison, and the remaining cells were stimulated with IL-2 and irradiated aAPCs bearing no cytokine (Clone 9), mbIL15 (Clone 4), mbIL21 (Clone 9.mbIL21), or IL-15 fused with a linker to IL15Rα (Clone 27). (A) Telomere length of the NK cells was determined after 7 days by flow cytometry after hybridization with FITC-labeled PNA probe for the TTAGGG telomeric repeat sequence. Mean fluorescence of the NK cells was normalized to the reference cell line CEM-1301, and the telomere length of expanded NK cells was normalized to that of fresh NK cells for each individual donor. A 1-way ANOVA with Dunnett's multiple comparisons test was applied to compare each aAPC to Clone 4. (B) NK cells from four additional donors stimulated weekly for 21 days prior to assessing telomere length, and again normalized to that of fresh cells from each donor. P value shown for two-tailed *t-*test.

Since expansion with mbIL15 was previously shown to be limited by telomere shortening [70] and we had seen significantly reduced senescence when NK cells were expanded with mbIL21, we questioned whether the telomeres of expanded NK cells differed depending on the cytokine support they received. To ensure that the telomere shortening previously shown was not a result of inadequate signaling of the mbIL15 because of lacking higher-affinity binding provided by the sushi domain during transpresentation by IL15Rα, we also constructed an aAPC (Clone 27) expressing the IL15∶IL15Rα fusion (IL-15 fused with a linker to IL-15Rα). We then determined the telomere lengths of fresh NK cells seven days after stimulation with Clone 4, Clone 27, Clone 9, or Clone 9.mbIL21, as compared to the telomere length of freshly-obtained unstimulated NK cells. IL-2 or mbIL15-expanded NK cells showed a nonsignificant trend towards slightly longer mean telomere lengths during this first week, whereas mbIL21 caused a significant increase in the mean telomere lengths of expanded NK cells (Figure 6A) despite having the greatest amount of proliferation. We then assessed the telomere lengths of 21-day expanded NK cells compared to fresh NK cells. Despite a mean 58-fold greater expansion at this timepoint mbIL21-expanded NK cells had increased their telomere lengths by a mean of 11.69%, where expansion with mbIL15 resulted in a mean 11.85% decrease in telomere length (Figure 6B).

### mbIL21-expanded NK cells have high cytotoxicity and cytokine secretion and retain KIR inhibition

**Figure 7:**
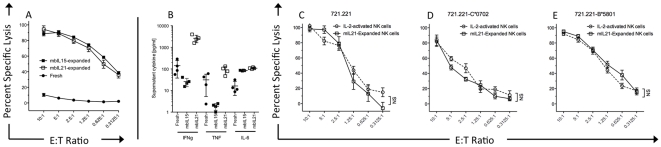
Direct cytotoxicity and cytokine secretion of fresh NK cells, mbIL15-expanded, and mbIL21-expanded NK cells. NK cells were purified from four normal donor buffy coats and assessed directly, and then retested after being expanded for 21 days with Clone 9.mbIL21 and Clone 4 (mbIL15) (with CD3 depletion on day 14). NK cells were assessed for cytotoxicity against 721.221 (A) or cytokine secretion in response to K562 (B). NK cells from another four donors were purified and cryopreserved, and expanded with Clone 9.mbIL21 and then cryopreserved. Fresh (circles) and expanded (squares) NK cells were then thawed and activated in parallel for 24 h in 50 IU/mL IL-2, and then tested for cytotoxicity against HLA^null^ 721.221 targets (C), C*0702-transduced (Group C1) 721.221 targets (D), or B*5801-transduced (Group Bw4) 721.221 targets (E). All data points shown are mean +/− SEM of the means of four donors tested in triplicate.

With high expression of granzyme B and perforin (Figure 5), we expected NK cells expanded with mbIL21 to be cytotoxic against tumor targets. However, others have reported that IL-21 has an inhibitory effect on some NK cells [50], [56] and may reduce expression of activating receptors [71], and the high expression of inhibitory KIR on expanded NK cells (Figure 4) led us to question whether they had enhanced inhibition by KIR ligands compared to that of non-expanded NK cells. We used the 721.221 cell line, which is HLA-negative, and two variants of this cell line transduced to express a group C1 (HLA-C*0702) or Bw4 (B*5801) KIR ligand as previously described [58], and assessed cytokine secretion in response to the parental K562 cell line. We found that the cytolytic ability of the mbIL15- or mbIL21-expanded NK cells toward the 721.21 cell line were similar and much greater than that of freshly isolated NK cells (Figure 7A). In contrast, mbIL21-expanded NK cells expressed significantly higher amounts of IFN-γ (mean 2629 pg/ml vs 26 pg/ml) and TNF-α (mean 90 pg/ml vs. 2 pg/ml) than mbIL15-expanded NK cells (Figure 7B). When cryopreserved mbIL21-expanded NK cells were recovered overnight with IL-2, they exhibited similar cytolysis of each of the KIR-ligand-expressing 721 targets (Figure 7C–E) as cryopreserved unexpanded NK cells recovered overnight with IL-2, suggesting that their responses to KIR ligands were unaffected by expansion.

### NK cells expanded with mbIL21 retain cytotoxicity against a wide variety of tumor cell lines and participate in ADCC

**Figure 8:**
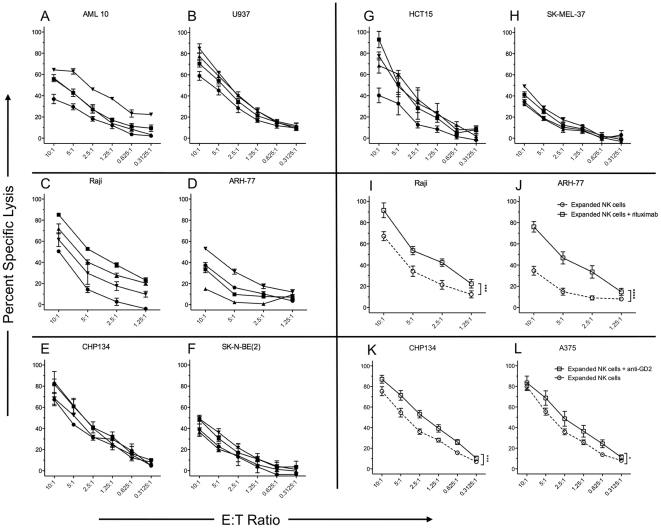
Direct and antibody-dependent cellular cytotoxicity of mbIL21 expanded NK cells toward tumor cell lines. NK cells were expanded for 21 days from four normal donors with CD3 depletion on day 14, and then cryopreserved in aliquots. NK cells were thawed and rested for 24 h in 50 IU/mL IL-2, and then tested for cytotoxicity against the indicated tumor cell lines using the calcein release assay. Assays were performed in triplicate, and results are shown as mean +/− SD for each donor against representative AML (A, B), B-cell (C, D), neuroblastoma (E, F), colon carcinoma (G), and melanoma (H) tumor cell lines. Squares, circles, triangles, and inverted triangles correspond to the expanded NK cells from the same four independent donors in each panel. I–L, expanded NK cells were tested for direct cytotoxicity (circles) or ADCC (squares) against B cell tumors (I, J) using anti-CD20 antibody (rituximab), or neuroblastoma (K) and melanoma (L) using anti-GD_2_ antibody (14G.2a). Data shown are mean +/− SEM of each donor tested in triplicate (A–H) or of the means of all four donors (I–L).

We next evaluated the cytotoxicity of mbIL21-expanded NK cells from four donors against a panel of human tumor cell lines representing myeloid, lymphoid, sarcoma, and carcinoma tumor types (Figure 8). In order to allow the most consistent comparisons between multiple donors and across multiple cell targets, we cryopreserved the NK cells after expansion. Unlike most reports of primary NK cells which cryopreserve poorly, we found that the expanded NK cells retained high viability 24 h after thawing with low-dose IL-2 rescue (>90%, data not shown). We found moderate to high cytotoxicity against all cell lines tested, with high sensitivity of AML (Figure 8 A, B) and neuroblastoma (Figure 8 E, F) as is commonly described.

To evaluate the interactions between NK cells and tumor targets during the killing process, we performed video microscopy of expanded NK cells co-cultured with calcein-labeled CHP-134 neuroblastoma cells as targets (Movie S1). The apparent contact time between NK cells and targets ranged from 1 to 8 frames (4 to 32 minutes), and some NK cells appeared to kill several target cells in rapid succession.

Since our expanded NK cells were CD16^bright^ by flow cytometry, we also hypothesized that they would participate in ADCC and exhibit enhanced cytotoxicity to antibody-labeled targets. Despite already exhibiting high baseline cytotoxicity towards Raji, the expanded NK cells showed a significant increase in cytotoxicity with the addition of rituximab (Figure 8I). Moreover, the ARH-77 cell line, which is reported to be rituximab-resistant, also showed significant sensitivity to rituximab-mediated ADCC (Figure 8J). Despite poor reported affinity of human CD16 for murine IgG2a, the 14.G2a anti-GD_2_ monoclonal also mediated significant ADCC by expanded NK cells when tested against GD_2_-expressing neuroblastoma and melanoma cell lines (Figure 8K–L).

## Discussion

Pilot studies of adoptive transfer of IL-2-activated NK cells have demonstrated responses in a wide variety of malignancies (reviewed in reference [72]). However, advances in adoptive immunotherapy with NK cells have been hindered by the lack of robust, clinically-relevant methods for NK cell expansion. There have been important recent advances in this regard, but the methods describing the greatest fold expansion to date have nonetheless been limited by NK cell senescence resulting from telomere shortening. Here we describe the introduction of a membrane-bound chimera of IL-21 to K562-based aAPCs, abrogating the need for clinical-grade production of cytokines other than IL-2 and enabling sustained *ex vivo* expansion of NK cells with increased telomere lengths and greatly reduced senescence.

IL-2, IL-15, and IL-21 are all members of the common γ-chain receptor family and their effects on NK cells are well-described. IL-21 is known to signal primarily through the STAT3 component of the JAK/STAT pathway with very little STAT5 involvement, whereas IL-15 signals primarily through STAT5. STAT3 is known to be an activator of human telomerase reverse transcriptase (hTERT) [73], and NK-cell senescence from mbIL15-mediated expansion can be restored with hTERT gene modification [70], though the cells become cytogenetically unstable. We found that NK cells expanded with mbIL21 have longer telomeres than those expanded with mbIL15. NK cells expanded with mbIL15 had shorter telomeres than those expanded with soluble IL2 alone, but this was likely a result of increased proliferation, not decreased hTERT activity. Increased TERT expression may also increase survival through mechanisms independent of telomere shortening [74]. Thus, the influence of IL-21 and STAT3 signaling on NK cell apoptosis should be explored. Adoptive transfer of lymphocytes with longer telomeres, e.g. “young TIL”, may have a significant advantage in the clinical setting through their greater potential for *in vivo* expansion [75].

IL-21 has both immunosuppressive and immunostimulatory effects on anti-tumor immunity [76]. IL-21 and STAT3 signaling are dispensable in some murine models of immunity [77], [78] and essential in others [79]. IL-21 has a differential effect on malignant lymphoid cell lines of B cell and T cell origin- it induces growth stimulation in B cell lines, but in T cell lines it promotes apoptosis through increased Bax, caspase-3, and caspase-8, but decreased Bcl-2 expression [80]. However, the direct role of IL-21 and STAT3 activation on NK cells is less well defined and somewhat controversial. IL-21 promotes differentiation of cord blood hematopoietic stem cells into NK-like lymphocytes when combined with Flt3-L and stem cell factor (SCF) [81]. IL-15 and IL-21 are synergistic for cytokine secretion [50], [82], cytotoxicity [50], proliferation [27], [83], and KIR expression [27], but soluble IL-21 severely blocks the IL-15-induced proliferation of murine NK cells [50], [51], in sharp contrast to our findings of enhanced proliferation when mbIL21 was added to mbIL15. Though IL-21 has been reported to downregulate NKG2D in NK cells [71], we found no difference in NKG2D expression between NK cells expanded with mbIL15 and mbIL21 in our system.

We also found that expression of CD160 mRNA transcripts was increased in mbIL21-expanded NK cells compared to those expanded with mbIL15, and confirmed increased expression of CD160 by flow cytometry (not shown). The transmembrane form of CD160 has been identified as a receptor selectively expressed on activated NK cells [84], [85]. K562 may express small amounts HLA-C, the physiologic ligand for CD160, and engagement of CD160 results in distinct cytokine secretion profile of IL-6, IFN-γ, and TNF-α [86]. Thus, increased CD160 expression in mbIL21-expanded NK cells may effect proliferation, cytokine profiles, or anti-tumor activity.

NK cells play a crucial role in antibody immunotherapy through ADCC, but expansion of NK cells has been sufficiently problematic in the past that others have proposed adoptive immunotherapy with expanded γδ T cells [87] or CD16 gene-modified [88] T cells as alternative approaches to enhancing ADCC of anti-cancer antibodies. IL-21 can enhance cytotoxicity to antibody-coated tumor targets [89], consistent with our finding of high expression of CD16 and functional ADCC in mbIL21-expanded NK cells. The relatively minor increase in cytotoxicity provided by ADCC with anti-GD_2_ mAb may be a result of the low affinity of this murine isotype for human CD16, the high baseline cytotoxicity of expanded NK cells for these targets, or that tumor cells may acquire resistance to ADCC through expression of their own FcRγIIb [90]. Enhancing ADCC by adoptive transfer of such cells may overcome some mechanisms of resistance, as we demonstrated for rituximab. Techniques for enhancing antibody-binding to CD16, the low-affinity Fcγ receptor, will further improve this approach [91].

In addition to wide variation in NK cell content of peripheral blood, there is wide variation in the proportion that are CD56^hi^, accounting for 3.5–37.5% of total NK cells [5]. In peripheral blood, CD56^hi^CD16^neg^ NK cells are understood to represent an immature stage of NK cell development characterized by increased proliferative potential [92] and longer telomeres [93], and these populations are differentially effected by IL15 and IL21 [27]. However, this population persists in both mbIL15 and mbIL21-expanded cultures, and despite the fact that mbIL21-epxanded cells have increased proliferation and longer telomeres, the proportion of CD56^hi^CD16^pos^ is not different between the two membrane-bound cytokines. In addition, others have demonstrated that CD56^dim^CD16^pos^ NK cells do not have significant anti-tumor cytotoxicity, which we find is not true of *ex vivo* expanded NK cells.

The critical ligands provided by K562 for NK cell expansion have not been clearly defined. In *ex vivo* NK cell expansion driven by allogeneic feeder cells, proliferation is enhanced in cells expressing KIR for which the self-HLA ligand is missing [94]. However, HLA engagement by CD160 promotes optimal cytokine release by NK cells [86]. Since K562 has only very low expression of HLA-Cw3 [95], this may explain the slight repertoire bias for KIR2DL2,3-expressing cells when expanded on K562 aAPCs. This raises the possibility that intentional skewing of the KIR repertoire during expansion could be accomplished by additional engineering of the aAPCs to express desired HLA haplotypes for KIR-mediated inhibition and/or licensing. In addition, high-dose IL-2 has been shown to preferentially expand KIR^neg^ NK cells. In contrast, expansion with K562 aAPCs results in NK cells with KIR diversity and high KIR expression. However, despite high KIR expression, we saw no difference in responses to KIR-ligand-engineered targets as compared to unexpanded NK cells. This data, along with our data regarding CD56^hi^CD16^pos^ subsets, suggests that the immunophenotype of *ex vivo* expanded NK cells may not be predictive of function when compared to known peripheral blood subpopulations. We were able to expand our NK cells with relatively low concentrations of IL-2 and observe high viability and sustained function after cryopreservation. Such properties may enable production of qualified cryopreserved lots for repeated doses in adoptive immunotherapy, and may allow reduced *in vivo* IL-2 support.

NK cells have demonstrated anti-cancer effect in clinical trials. However, their widespread use is hampered by the ability to generate large numbers of these cells *ex vivo*. Currently, most investigators rely on leukapheresis, commercially-available paramagnetic beads, and clinical large-scale magnetic separation devices to generate NK-cell products, a process that is expensive and results in cells with either sub-optimal purity (CD3-depletion alone) or potency (CD3 depletion and CD56 selection). Because most methods of NK-cell expansion also result in some degree of non-specific T-cell expansion, and contaminating T cells in the clinical NK-cell product should be avoided because of the attendant risk of GvHD (particularly in the setting of haploidentical donors), we employed T-cell depletion at various stages of the expansion process. To avoid the need for large-scale CD3-depletion of the final product (which would be very costly at higher cell doses [96]), we first attempted selection of NK cells prior to expansion, similar to that described by Berg [41], and found no significant effect on the proliferation of the NK cells. Early timing of T-cell depletion will enable pure NK cell products to be propagated with aAPCs to clinically-appealing numbers from a single venipuncture sample, circumventing the need for apheresis or large-scale depletion. To achieve a GMP-compliant system of lymphocyte expansion that minimizes reliance on external sources of GMP-grade cytokines, our group has focused on generating aAPCs with membrane-bound cytokines [60], [61], [97], [98]. The method we used herein requires only low concentrations of IL-2 (50 IU/mL). In addition, we have achieved the high expansion rate described without the use of specialized culture media required by other systems. All of these features should reduce the costs associated with NK cell adoptive immunotherapy.

Adoptive transfer of NK cells after lymphodepleting chemotherapy has shown promise in a variety of cancer settings, but the greatest potential may be realized in the post-transplant setting, as early and vigorous NK cell reconstitution may reduce GvHD, reduce infectious complications, and increase anti-cancer activity in the MRD setting. Though the lack of adequate numbers of NK cells for adoptive immunotherapy is widely viewed as a hindrance to advancing the field, there is still much controversy regarding expansion methods and whether *in vitro* or *in vivo* expansion is superior [99], [100]. The increased expansion potential afforded by mbIL21 should eliminate the need for donor apheresis, enable cryopreservation and single lot-release testing of multiple infusion aliquots for repeated dosing, and provide sufficient cells from a single venipuncture to achieve total cell doses in excess of 10^10^/kg, enabling clinical trials that address these questions.

## Supporting Information

Table S1 **Transcription of a selected set of immune-related genes was performed using the nanoString platform by nCounter, with a custom designed probeset.** The common gene name, the accession number and region for the sequence against which the target gene probe was designed, and the unique capture/detection sequences for each gene are listed as indicated.
(XLS)

Movie S1 **Video fluorescent microscopy of NK cell lysis of neuroblastoma targets.** CHP134 neuroblastoma targets were loaded with calcein dye as described. NK cells were added to target cells at a 5∶1 E∶T ratio, and fluorescent and brightfield images were acquired at 4-minute intervals using the Nikon BioStation. Sudden loss of fluorescence indicates a loss of cell membrane integrity associated with perforin release by NK cells. Total duration of the movie file spans 2 hours 40 minutes.
(MOV)
